# Health impact of US military service in a large population-based military cohort: findings of the Millennium Cohort Study, 2001-2008

**DOI:** 10.1186/1471-2458-11-69

**Published:** 2011-01-31

**Authors:** Tyler C Smith, Isabel G Jacobson, Tomoko I Hooper, Cynthia A LeardMann, Edward J Boyko, Besa Smith, Gary D Gackstetter, Timothy S Wells, Paul J Amoroso, Gregory C Gray, James R Riddle, Margaret AK Ryan

**Affiliations:** 1 Center for Deployment Health Research, Naval Health Research Center, 140 Sylvester Rd., San Diego, CA 92106-3521, USA

## Abstract

**Background:**

Combat-intense, lengthy, and multiple deployments in Iraq and Afghanistan have characterized the new millennium. The US military's all-volunteer force has never been better trained and technologically equipped to engage enemy combatants in multiple theaters of operations. Nonetheless, concerns over potential lasting effects of deployment on long-term health continue to mount and are yet to be elucidated. This report outlines how findings from the first 7 years of the Millennium Cohort Study have helped to address health concerns related to military service including deployments.

**Methods:**

The Millennium Cohort Study was designed in the late 1990s to address veteran and public concerns for the first time using prospectively collected health and behavioral data.

**Results:**

Over 150 000 active-duty, reserve, and National Guard personnel from all service branches have enrolled, and more than 70% of the first 2 enrollment panels submitted at least 1 follow-up survey. Approximately half of the Cohort has deployed in support of operations in Iraq and Afghanistan.

**Conclusion:**

The Millennium Cohort Study is providing prospective data that will guide public health policymakers for years to come by exploring associations between military exposures and important health outcomes. Strategic studies aim to identify, reduce, and prevent adverse health outcomes that may be associated with military service, including those related to deployment.

## Background

Soon after the end of the 1991 Gulf War, veterans began reporting symptoms and illnesses they perceived to be possibly related to exposures during deployment. However, research on illnesses related to the 1991 Gulf War was hampered by the non-availability of objective measurements on exposures at the individual level, a lack of baseline health data, and an inability to adequately control for potential confounding factors. Baseline health data prior to potential military exposures are critical to appropriately evaluate associations between deployment-related exposures and subsequent health outcomes. Previous research involving cross-sectional and case-control study designs suffered from recall bias [1], inability to assess temporal association between exposure and outcome, and lack of the ability to evaluate incident cases of illness or injury and attributable risk. Thus, the importance of conducting prospective research on questions relevant to military veterans was clear. Therefore, public health experts and other stakeholders recommended that the Department of Defense (DoD) establish a cohort to prospectively study both short- and long-term health effects of military service [1], and this effort was subsequently endorsed by congress [2,3]. The Millennium Cohort Study was designed and launched to address this need [3,4].

Health status is assessed at baseline using self-reported questionnaire data linked to supplemental data from various DoD administrative and health databases. Follow-up data are collected every 3 years through postal or Web-based surveys with periodic queries of the same electronic databases. The launch of the Millennium Cohort Study in July 2001 occurred just prior to terrorist attacks on the United States on September 11, 2001, and the start of military mobilization and engagement in multiple combat theaters. Consequently, a significant proportion of study participants have experienced military deployment, and the stage was set to better understand any health consequences of these deployments.

## Methods

### Study Population

The Millennium Cohort Study is a population-based study consisting of participants drawn from a randomly selected sample of all US military service members on rosters as of October 2000 (Panel 1), October 2003 (Panel 2), and October 2006 (Panel 3) [4,5]. Panel 1 was a probability-based sample of the entire military population. To ensure adequate statistical power to detect rare health outcomes in smaller subgroups of this first enrollment panel, women, those who had past deployment experience, and those who were in the reserves or National Guard were oversampled. Panels 2 and 3 were designed to include newer accessions. Panel 2 included personnel with 1 to 2 years of military service and panel 3 included personnel with 1 to 3 years of military service. Marine Corps members and women were oversampled in both of these panels. Over 150 000 service members have voluntarily consented and completed baseline questionnaires since the first wave of invitations in 2001 (July 2001-June 2003; n = 77 047), 2004 (Panel 2 enrolled between June 2004-February 2006; n = 31 110), and 2007 (Panel 3 enrolled between June 2007-December 2008; n = 43 440), resulting in a 34% cumulative baseline response rate. Over 70% of the first 2 panels have submitted at least 1 follow-up questionnaire.

Demographic and military personnel data from the Defense Manpower Data Center (DMDC) were obtained for each cohort member. These data include sex, age, marital status, race/ethnicity, education, service branch (Army, Air Force, Navy/Coast Guard, and Marine Corps), service component (active duty, Reserve/National Guard), occupation, and military pay grade (officer, enlisted).

### Millennium Cohort Foundation Studies

A number of early studies were planned and executed to establish the representativeness of the Cohort, understand response bias, assess reliability in reporting, ascertain mortality, compare Web- and paper-based responses, and validate self-reported health outcomes with automated health information. These foundation analyses have documented a cohort representative of the general US military, data quality and reliability which are excellent, and little to no evidence of response bias [4,6-15].

In order to examine possible response bias, prior hospitalization and ambulatory care experience of responders was compared with that of nonresponders [6]. Findings demonstrated that, although there were a few minor differences in health care use, prior health care use did not appear to influence voluntary participation in the Millennium Cohort Study. In addition, overall baseline prevalence of mental disorders and functional health compared favorably with other civilian and military populations [12,15]. Other foundation studies have addressed characteristics of subgroups, such as early refusers and early consenters [14] and Internet and paper responders [10], and demonstrated similarities in demographic and baseline health characteristics. Both internet and paper responders completed, on average, 98% of the survey questions with no indication of fatigue bias. Inclusion of a Web-based survey increased data quality and quantity [10,16], reducing the opportunity for participants to provide unusable data, with minimal likelihood of enrolling a nonrepresentative group. Added benefits included an informative Web site intended to foster a relationship with cohort members and to provide a means for updating contact information [17].

The degree of nonrandom agreement between self-reported Millennium Cohort survey data and electronic records has been examined using Kappa statistics, where a kappa between 0.8-1.0 indicates "greater than substantial agreement," between 0.6-0.8 "substantial agreement," between 0.4-0.6 "moderate agreement," between 0.2-0.4 "fair agreement," and between 0.0-0.2 "slight or poor agreement" [18]. Strong reliability was found for anthrax vaccination (κ =.80), smallpox vaccination (κ = 0.62), deployment status (κ = 0.81), and deployment frequency (κ = 0.72) [9,11,13]. While a Kappa value of 0.81 can be interpreted as greater than substantial agreement [18], one might have expected an even higher Kappa for deployment status (agreement on whether or not a deployment in support of the operations in Iraq or Afghanistan occurred during the past 3 years). Perhaps some participants were self-reporting a deployment that occurred during a different time period or to a location not listed, or in support of another operation. It is also possible that a participant may have been a member of a special operations unit and therefore unable to reveal their deployment status. Additionally, in a study of female Cohort members, self-reported occupations were moderately to highly reliable when compared with electronic occupational data (κ =.65) [8]. Finally, an examination of internal consistency and test-retest reliability of standard health assessment instruments embedded in the study questionnaire found high internal consistency for 14 of 16 health components [7]. Only alcohol misuse as measured by the PHQ and the CAGE questionnaires showed low internal consistency, potentially reflecting variability in reporting by individuals who perceive their behavior as problematic. Substantial test-retest stability was observed for variables not subject to change such as whether or not the subject was a twin, as well as variables less likely to change frequently over time, including marital status or education, while moderate stability was found for more dynamic variables [7].

### Deployment and Deployment-Related Exposures

A major objective when designing the Millennium Cohort Study was to describe deployment experiences and exposures among US service members and relate them temporally to subsequent health outcomes. Special emphasis was placed on obtaining information on vaccines, environmental exposures, and combat-related experiences.

Electronic deployment data are obtained from DMDC and include in- and out-of-theater dates for current operations. The study questionnaire also uses 24 country and sea codes to assess self-reported date and location of deployment since 2001.

Table 1 illustrates the demographic and military characteristics of Millennium Cohort participants by enrollment panel and deployment experience. The demographic makeup of all 3 panels, consistent with the military in general, is composed of a higher proporti on of men; enlisted, active-duty, and Army personnel; and personnel in occupations other than combat. By design, Panel 1 consists of a cross section of the entire military compared with Panels 2 and 3, consisting of new accessions. This table also highlights that deployed participants are representative of the total population in their respective panels, except that a higher proportion of individuals with deployments to Iraq and Afghanistan between 2001 and 2007 were male, slightly younger, active duty, and combat specialists.

**Table 1:** Characteristics of Millennium Cohort Study Participants by Panel and Deployment Experience

	Panel 1			Panel 2		Panel 3	
	
	All Participants	**2001-2009 Deployment Experience**^**b**^	**Previous Deployment Experience**^**c**^	All Participants	**2001-2009 Deployment Experience**^**b**^	All Participants	**2001-2009 Deployment Experience**^**b**^
	N = 77 047	N = 35 564	N = 28 897	N = 31 110	N = 19 598	N = 43 440	N = 27 777
	**n (%)^a^**	**%**	**%**	**n (%)^a^**	**%**	**n (%)^a^**	**%**

Gender							
Male	56 415 (73.2)	80.5	86.7	19 167 (61.6)	68.0	27 941 (64.3)	71.1
Female	20 632 (26.8)	19.5	13.3	11 943 (38.4)	32.0	15 499 (35.7)	28.9
Birth year							
Before 1960	16 652 (21.6)	13.4	18.0	226 (0.7)	0.6	69 (0.2)	0.1
1960-1969	29 177 (37.9)	39.3	45.7	1678 (5.4)	4.4	735 (1.7)	1.4
1970-1979	26 672 (34.6)	40.6	35.3	9934 (31.9)	29.7	6820 (15.7)	15.3
1980 or later	4546 (5.9)	6.7	1.0	19 272 (62.0)	65.4	35 816 (82.4)	83.3
Military rank							
Enlisted	59 318 (77.0)	76.8	78.0	27 482 (88.3)	88.5	38 455 (88.5)	88.5
Officer	17 729 (23.0)	23.2	22.0	3628 (11.7)	11.5	4985 (11.5)	11.5
Service component							
Reserve/National Guard	33 157 (43.0)	37.3	25.6	12 459 (40.1)	34.9	8992 (20.7)	17.0
Active duty	43 890 (57.0)	62.7	74.4	18 651 (60.0)	65.1	34 448 (79.3)	83.0
Service branch							
Army	36 481 (47.4)	49.3	38.5	14 995 (48.2)	54.9	15 798 (36.4)	40.6
Air Force	22 357 (29.0)	30.2	37.5	8276 (26.6)	23.5	12 918 (29.7)	26.8
Navy	14 268 (18.5)	15.3	19.8	5263 (16.9)	14.2	7922 (18.2)	15.0
Marine Corps	3941 (5.1)	5.1	4.3	2576 (8.3)	7.4	6802 (15.7)	17.5
Military occupation							
Combat specialists	15 425 (20.0)	23.7	24.0	4868 (15.7)	18.9	6591 (15.2)	18.4
All others	61 622 (80.0)	76.3	76.0	26 242 (84.4)	81.1	36 849 (84.8)	81.6

^a ^Percentages rounded up and may not sum to 100.

^b ^Deployed in support of the Global War on Terrorism between September 1, 2001, and August 31, 2007.

^c ^Deployed to the 1991 Gulf War or to Bosnia, Kosovo, or Southwest Asia between 1998 and 2000.

Among preventive measures used to counter threats of biological warfare agents, most deployed US service members receive anthrax and smallpox vaccinations. The Anthrax Vaccine Immunization Program began in the late 1990s, while the smallpox vaccination program began in 2001 [19,20]. In addition to these electronic vaccination data, questionnaires ask respondents whether they have ever received the anthrax vaccine, and if so, the number of shots they received. Beginning with the 2004-2006 survey, the questionnaire also asked whether members received the smallpox vaccine after 2001. These data have been used to assess differences between self-reported vaccination history and electronic records [11,13], as well as to identify any associated health differences for concordant or discordant groups [21].

Questions from the National Health Survey of Gulf War Era Veterans [22] were incorporated into the Millennium Cohort questionnaire to ascertain combat-related exposures and experiences. Higher rates of posttraumatic stress disorder (PTSD) among US combat infantry members following deployment to Iraq compared with those deployed to Afghanistan or nondeployed have been reported [23]. This observation led to a hypothesis that combat experiences while deployed were more influential predictors of PTSD than deployment per se without exposure to combat. Data from the Millennium Cohort support increased risk for both PTSD and depression among study participants who deployed and reported combat exposure [24-27]. To increase our understanding of any health effects associated with combat exposures, the 2007-2008 questionnaire was amended to incorporate an additional 13 questions on combat-related experiences. In addition, questions on significant injuries that included head trauma were also added to support investigations of mild traumatic brain injury (concussion) [28].

Because deployment may lead to increased risk of exposure to environmental hazards, such as exposure to pesticides [29,30], chemical munitions [31,32], and other environmental exposures, such as airborne pollutants [33], the questionnaire includes several items to support studies of health effects associated with environmental exposures. Separate questions relate to potential occupational hazards requiring the use of personal protective equipment, exposure to dermal hazards, depleted uranium, microwaves, or insecticides. Cohort members may choose to provide an open-ended response to share any other physical or psychological exposure of concern.

### Completed, Ongoing, and Future Analyses

The longstanding and continued combat operations in Iraq and Afghanistan have fueled ongoing concerns among veterans and the general public over unknown exposures and potential long-term health consequences of serving in the military and, in particular, of deployment. Mental health disorders and more subtle physical sequelae may affect both short-term and long-term functional capacity and quality of life in troops returning from deployment. Studies have reported significantly higher rates of mental health disorders, such as PTSD, major depression, and alcohol misuse, after deployment in support of combat operations in Iraq and Afghanistan, as well as the 1991 Gulf War and the Vietnam War [23,34-40]. Findings from the Millennium Cohort Study describing associations between deployment and mental health and behavioral outcomes have already informed policymakers who strive to address the needs of veterans [15,24-27,41-45]. Results from the Millennium Cohort Study were also cited in a recently published Institute of Medicine report that identified physical and mental health, as well as readjustment needs, of current and former service members deployed to Iraq or Afghanistan [46]. Military occupational and deployment-related stress may manifest in maladaptive coping mechanisms, including smoking, particularly among those reporting acute and chronic stress [47-49]. Over an average follow-up of 2.7 years, the prevalence of cigarette smoking increased in Millennium Cohort Panel 1 members when compared with baseline measures [50]. The increase in smoking was greater among those who deployed than those who did not, with those deploying multiple times or reporting combat exposures at greatest risk. Increases in smoking postdeployment were predominantly explained by recidivism among former smokers who had successfully quit prior to deployment. Future investigations will help determine whether newly initiated or reinitiated smoking following deployment is temporary or persists over time.

Previous cross-sectional and serial cross-sectional studies have reported alcohol misuse among personnel returning from combat operations in Iraq and Afghanistan [34,51]. Alcohol use before and after deployment was also investigated using longitudinal data from this Cohort [43]. Findings revealed that Reserve/Guard personnel who deployed and reported combat exposures were at increased risk for newly reported heavy weekly drinking, binge drinking, and alcohol-related problems at follow-up when compared with nondeployed personnel. Only an increased risk of newly reported binge drinking at follow-up was observed among active-duty personnel who deployed and also reported combat exposures. As we continue to measure alcohol use among Cohort participants over time, we will better understand how combat deployments affect alcohol use.

Investigation of deployment and new-onset disordered eating and changes in weight between baseline and follow-up surveys showed no differences among men and women who deployed, with or without combat exposures, and those who did not deploy [25]. When deployers were examined separately, women with combat exposures were more likely to develop an eating disorder between baseline and follow-up compared with those who did not experience combat. Additionally, deployment status did not appear to be associated with weight change between baseline and follow-up. With the increasing trends of obesity in the United States, continued evaluation of weight changes over time, including assessment for comorbid conditions and behavioral characteristics, has great public health implications.

In addition to changes in health risk behaviors possibly associated with deployment-related stressors, psychiatric disorders, such as depression and PTSD, have also received significant attention. To investigate depression among Millennium Cohort participants, the study questionnaire includes a standard self-administered clinical instrument to evaluate mental disorders, the Primary Care Evaluation of Mental Disorders Patient Health Questionnaire [52], and self-reported history of provider-diagnosed depression. After adjustment, deployment in support of the conflicts in Iraq and Afghanistan was associated with new-onset depression [24]. Specifically, combat-deployed men and women were at increased risk of new-onset depression compared with nondeployed men and women. Conversely, deployment without combat exposures led to decreased risk of new-onset depression compared with those who did not deploy.

PTSD symptoms have been reported among as many as 30% of veterans following service in Vietnam and in as many as 10% of personnel returning from the 1991 Gulf War [36,53-58]. More recent studies using cross-sectional data have indicated that between 6% and 20% of returning service members screen positive for PTSD [23,59], and 22% of Iraq and Afghanistan veterans entering the VA health care system were diagnosed with PTSD [60]. The Millennium Cohort, a longitudinal study with approximately 50% of its participants deployed in support of the wars in Iraq and Afghanistan, is well positioned to investigate new onset, persistence, and resolution of PTSD symptoms among veterans of the current deployments. Recently published findings using Millennium Cohort data suggest a baseline 2% prevalence of undiagnosed PTSD [41], a 3-fold increase in new-onset PTSD symptoms or diagnosis among deployed military personnel reporting combat exposures [27], and a 2-fold higher risk of new-onset PTSD symptoms in both female and male combat deployers who reported assault prior to deployment [26]. Additional findings from analyses of Millennium Cohort data suggest that a vulnerable population at increased risk of PTSD may be identified through predeployment screening [42].

Ongoing prospective investigations are critical to increasing our understanding of PTSD and include exploring the time course of new-onset and persistent PTSD. Furthermore, identifying and testing potential interventions to speed recovery and promote resilience in military members facing new challenges is vitally important. Complementary and alternative therapies, physical exercise, and identification and treatment to minimize effects of mild to severe traumatic brain injury may mitigate symptoms of PTSD. The 2007-2008 survey cycle added a third longitudinal data point for further investigation of new onset, persistence, and resolution of PTSD symptoms and offer a better understanding of resilience factors among cohort members.

Table 2 provides a summary comparison of new-onset behavioral and mental health outcomes among Panel 1 participants between baseline and first follow-up surveys. Across all outcomes, the highest proportions were observed among individuals deployed with combat exposures. It is also notable that smoking recidivism and new-onset binge drinking affected the highest proportion of participants compared with other outcomes, especially in the youngest age group. Among mental health outcomes, new-onset depression was most frequently reported among Cohort members, with the largest proportion occurring among those deployed with combat exposures, women, and those in the youngest age group.

**Table 2:** New-onset^a ^behavioral and mental health outcomes in Millennium Cohort Panel 1 participants between baseline and first follow-up surveys

	Health Outcome						
	
	**Smoking**^**b**^		**Alcohol Use**^c^				
	New Smoking	Smoking Recidivism	Binge-Like Drinking	Problem Drinking	**Disordered Eating**^**d**^	**Depression**^**e**^	**PTSD**^**f**^
	%	%	%	%	%	%	%
Recent deployment experience^g^							
Nondeployed	1.4	28.7	18.2	3.7	2.9	5.0	2.3
Deployed without combat	1.5	35.3	20.9	3.2	2.4	2.8	1.4
Deployed with combat exposures	3.3	44.7	26.2	5.7	3.0	7.3	7.6
Gender							
Male	1.6	29.5	21.4	4.2	2.6	4.0	2.4
Female	1.7	36.1	15.3	3.0	3.3	8.0	3.8
Birth year							
Before 1960	0.5	16.6	12.8	2.6	2.5	4.0	2.2
1960-1969	1.1	29.3	19.7	3.0	2.6	4.4	2.3
1970-1979	2.4	49.0	24.4	5.5	3.3	6.2	3.5
1980 or later	8.2	64.0	42.5	10.1	3.6	9.1	5.4
Military rank							
Enlisted	2.1	34.3	21.4	4.5	3.0	5.8	3.3
Officer	0.7	20.0	14.4	2.4	2.4	3.0	1.4
Service component							
Reserve/National Guard	1.4	28.4	18.0	4.1	2.5	5.1	2.9
Active duty	1.7	33.6	20.4	3.7	3.1	4.9	2.7
Service branch							
Army	2.2	33.6	19.9	4.4	3.0	6.1	3.9
Air Force	1.0	27.2	17.4	2.6	2.3	3.6	1.3
Navy	1.1	29.9	19.9	3.8	3.0	4.6	2.2
Marine Corps	2.4	38.1	23.8	7.6	3.2	4.1	3.0
Military occupation							
Combat specialists	1.4	30.5	21.4	4.2	2.7	3.6	2.4
All others	1.7	31.3	18.8	3.8	2.8	5.3	2.9

^a ^New-onset was defined among those with no baseline evidence of selected health outcomes.

^b ^New smoking defined among those with no lifetime history of prior smoking. Smoking recidivism defined among past smokers who resumed smoking between baseline and follow-up.

^c ^Binge-like drinking defined as drinking 5 or more drinks (for men) or 4 or more drinks (for women) on at least 1 day of the week, or any drinking of 5 or more alcoholic beverages on at least 1 occasion during the year. Problem drinking defined by endorsement of at least 1 item from the Patient Health Questionnaire (PHQ) for probable alcohol abuse [77].

^d ^Disordered eating defined by criteria from the PHQ[77].

^e ^Depression defined by criteria from the PHQ-9[77].

^f ^Posttraumatic stress disorder defined by DSM-IV criteria using the PTSD Checklist-Civilian Version with a score of 50 or more points [78].

^g ^Recent deployment was defined as a deployment in support of the wars in Iraq and Afghanistan that occurred between baseline and follow-up evaluations. Results exclude those who provided either baseline or follow-up information during deployment.

Increasing evidence suggests that a higher proportion of service members deployed to Afghanistan and Iraq are suffering from head and brain injuries compared with prior conflicts [61,62]. These head injuries are due, in part, to widespread use of improvised explosive devices by enemy combatants. The protection afforded by advanced body armor has allowed head-trauma patients to survive injuries that may have previously been fatal. Beginning in 2007, questions to obtain information about head and other injuries were added to the Millennium Cohort survey. These data will allow Millennium Cohort researchers to study health consequences associated with mild to severe traumatic brain injury, as well as other injuries sustained in relation to deployment and occupational exposures.

While injuries remain one of the most significant health problems of the armed services, it is the sequelae of injuries, especially musculoskeletal conditions resulting in permanent disability, that exact the greatest and most lasting toll on our service members [63]. Musculoskeletal injury-related disability has been growing rapidly over past decades, as has the cost of care [64], rehabilitation, and compensation for service-connected disability. The Millennium Cohort Study is uniquely poised to study the natural history of these chronic conditions among an aging cohort of former military service members with a variety of musculoskeletal injuries sustained while in service. Projects currently underway include assessment of service-related injuries associated with mental health pre- and post-deployment, as well as back pain related to deployment.

Military deployment has previously been identified as a risk factor for deaths due to external causes in general, and specifically due to motor vehicle crashes, following both the Vietnam War and the 1991 Gulf War [65-67]. The mechanisms for increased risk of fatal motor vehicle crashes among returning combat veterans from previous conflicts have yet to be fully understood. Several pathways for this increased risk have been proposed, including postdeployment mental disorders, such as PTSD; maladaptive coping mechanisms, such as alcohol misuse following stressful deployment experiences; increased risk-taking behaviors due to demanding training or combat experiences; and differentially distributed risk factors among those selected for deployment [66-68]. The Millennium Cohort offers a unique opportunity to prospectively investigate many of these potential risk factors.

As the Millennium Cohort Study moves toward the end of the first decade of data collection, longitudinal data points will be used to investigate chronic medical and psychiatric conditions and any short- or long-term impact of military deployment on the development or natural history of these conditions. Presence of chronic medical conditions will be identified using several different methods, including self-reported questionnaire responses and electronic DoD and Department of Veterans Affairs health care databases. Even after separation from military service, the Millennium Cohort survey instrument will be the main method of assessing new onset of chronic illnesses, since fewer than 20% of veterans use Veterans Affairs health care facilities. The younger age of the cohort limits our ability to identify exposure-health outcome associations for common causes of mortality, such as coronary heart disease or cancer, during early periods of follow-up. However, chronic conditions with earlier ages of onset should be detectable in relation to exposures of interest. These include recently investigated conditions such as asthma, hypertension, and diabetes, as well as planned future investigations of multiple sclerosis, inflammatory bowel disease, and infectious diseases such as hepatitis C. Similar to incidence estimates for diabetes from the CDC [69], newly self-reported diabetes in the Millennium Cohort was 3 per 1,000 persons [70], although risk for diabetes was not associated with combat exposures. Newly-reported hypertension, however, was found to be associated with reporting multiple combat exposures, especially witnessing death due to war [45]. Findings from the Cohort also showed that respiratory symptoms were significantly elevated among Army and Marine Corps personnel deployed in support of the operations in Iraq and Afghanistan, helping to more clearly define the complicated relationship between deployment and respiratory health outcomes [44]. Another complex yet important topic of interest will be reproductive health outcomes in this young adult population [71]. Future analyses using Cohort data will more clearly define the relationship between deployment and important health conditions and should shed considerable light on the question of increased disease risk in relation to common exposures experienced by US military members.

The current study is a population-based, prospective cohort design that allows for baseline and multiple follow-up assessments on the same individuals. The cohort design, large sample size, and ability to prospectively follow-up individuals for over 20 years make the Millennium Cohort arguably one of the most ambitious and challenging studies undertaken in the era of modern epidemiology [72,73]. However, a study of this size and complexity is not without limitations. The analyses use self-reported data from questionnaires. Nonetheless, the comprehensive survey instruments use standardized and validated questions when available and are administered at 3-year intervals (currently planned through 2022), although the full impact of ongoing conflicts and detection of more long-term health outcomes will likely require continued follow-up. Although clinical examinations to confirm self-reported symptoms and conditions were not planned as a part of this study, self-reported data from questionnaires can be linked with hospitalization discharge records that include diagnostic codes (providing an indication of disease severity). Previous comparisons between these self-reported and electronic data sources for specific medical conditions demonstrated general agreement [74].

## Conclusions

The concept for the Millennium Cohort Study arose from lingering postdeployment concerns following the 1991 Gulf War [1,75]. The population-based, prospective longitudinal design and launching of the baseline cohort just prior to combat operations in Iraq and Afghanistan were fortuitous and positioned the study to appropriately evaluate the temporal sequence of exposures and possible health outcomes resulting from military service and deployment without the substantial limitations of previous observational studies. Prolonged military operations in Iraq and Afghanistan since 2001 have focused concern on psychological morbidity and its long-term consequences. The Millennium Cohort Study has baseline data on physical and mental health status, as well as health-risk behaviors, from a large, population-based military cohort, with enrollment of the first panel beginning before the current military conflicts and follow-up data based on standard metrics for assessing new-onset health outcomes temporally related to exposures of concern [4,5]. The ability to identify high risk groups which are more likely to develop new-onset adverse mental health outcomes is of paramount importance and paves the way for more focused research in this area. Millennium Cohort Study findings on respiratory symptoms and psychological health outcomes including PTSD, depression, and substance abuse have and should continue to positively influence policymakers as they address the needs of veterans [12,15,24-27,41-43,50].

Perhaps most importantly, the Millennium Cohort Study will continue to play an important role in defining long-term health consequences of military occupational exposures, specifically during times of combat deployment. Such exposures include psychological and environmental stressors, medical interventions, and other occupational factors that are unique to military populations [76]. The health outcomes of interest, including chronic diseases, chronic sequelae of injury, and disability, are clearly of interest to veterans, the general public, and policymakers alike, making the potential impact of the Millennium Cohort Study far-reaching and unprecedented.

## Competing interests

The authors declare that they have no competing interests.

## Authors' contributions

TS originated the study and supervised all aspects of its implementation. IJ, CL, and BS assisted with analysis of data. IJ, TH, CL, EB, BS, GG, TW, PA, GG, JR, and MR assisted with design and interpretation of data, drafting and revision of the manuscript. All authors read and approved the final manuscript.

## Authors' information

At the time of this study, Tyler Smith, Isabel Jacobson, Cynthia LeardMann, and Besa Smith were at the Department of Defense Center for Deployment Health Research, Naval Health Research Center, San Diego, CA. Tomoko Hooper was with the Department of Preventive Medicine and Biometrics, Uniformed Services University of the Health Sciences, Bethesda, MD. Edward Boyko was with the Seattle Epidemiologic Research and Information Center, Department of Veterans Affairs Puget Sound Healthcare System, Seattle, WA. Gary Gackstetter was at Analytic Services, Inc. (ANSER), Arlington, VA. Timothy Wells and James Riddle were with the Air Force Research Laboratory, Wright-Patterson Air Force Base, OH. Paul Amoroso was with Madigan Army Medical Center, Tacoma, WA. Gregory Gray was with the Department of Environmental and Global Health, College of Public Health and Health Professions, University of Florida, Gainesville, FL. Margaret Ryan was with the Naval Hospital Camp Pendleton, Camp Pendleton, CA.

## Pre-publication history

The pre-publication history for this paper can be accessed here:

http://www.biomedcentral.com/1471-2458/11/69/prepub
